# Effects of Titanium Gypsum and Flue Gas Desulfurization Gypsum on the Hydration and Mechanical Properties of Anhydrite–Phosphogypsum-Based Supersulfated Cement

**DOI:** 10.3390/ma19061273

**Published:** 2026-03-23

**Authors:** Youquan Xie, Li Yang, Xiaodong Li, Jiaqing Wang, Yanbo Li, Hao Zhou, Yueyang Hu

**Affiliations:** 1 College of Materials Science and Engineering, Yancheng Institute of Technology, Yancheng 224002, China; youquanxie@163.com (Y.X.); lybybyb@126.com (Y.L.); 2 School of Civil and Safety Engineering, Wanjiang University of Technology, Ma’anshan 243031, China; 3 Nanjing Institute of Environmental Sciences, Ministry of Ecology and Environment of the People’s Republic of China, Nanjing 210042, China; lixiaodong@nies.org (X.L.); zhouhao@nies.org (H.Z.); 4 College of Civil Engineering, Nanjing Forestry University, Nanjing 210037, China; jiaqingw@njfu.edu.cn; 5 Anhui Conch Group Co., Ltd., Wuhu 241000, China

**Keywords:** supersulfated cement, flue gas desulfurization gypsum, titanium gypsum, hydration

## Abstract

Supersulfated cement (SSC) is an environmentally friendly cementitious material with a low clinker content, in which industrial byproduct gypsum serves as the sulfate source, thereby enabling the valorization of solid waste. The hydration process, pore structure, microstructure, and hydration products were investigated using paste samples by means of isothermal calorimetry, X-ray diffraction (XRD), thermogravimetric analysis (TG–DTG), Fourier transform–infrared spectroscopy (FT-IR), mercury intrusion porosimetry (MIP), and scanning electron microscopy (SEM), while compressive strength was evaluated using mortar specimens. Compared with ordinary Portland cement (OPC), SSC offers clear advantages in reducing energy consumption and greenhouse gas emissions. In this study, the effects of titanium gypsum (TG) and flue gas desulfurization gypsum (FGD) on the hydration behavior, fluidity, mechanical properties, and microstructural evolution of an anhydrite (AH)–phosphogypsum (PG)-based SSC were systematically investigated. The results indicate that the incorporation of 11% TG and FGD mitigates the strong sulfate environment caused by the rapid dissolution of soluble AH, thereby regulating the hydration process. As the proportion of TG and FGD increased, the cumulative heat release within 72 h gradually decreased. When AH was completely replaced, the cumulative heat release of TG4 and FG4 decreased by approximately 19.7% and 28.6%, respectively. TG and FGD exhibited opposite effects on the fluidity of SSC while both promoting strength development. Among all mixtures, TG2 and FG2 showed the best performance, with the highest 28-day compressive strengths of 50.15 MPa and 51.95 MPa, respectively. Microstructural analysis reveals that differences in particle size distribution and dissolution kinetics among gypsums governed the sulfate release characteristics and slag activation mechanisms, thus leading to distinct hydration pathways, pore structure evolution, and microstructural densification. This study provides a theoretical basis for the efficient utilization of various industrial byproduct gypsums and offers important guidance for the controllable design of SSC performance.

## 1. Introduction

With the intensification of global climate change and the continued advancement of green and low-carbon development strategies, the conventional cement industry is facing increasing challenges because of its high energy consumption and substantial greenhouse gas emissions. Moreover, the long-term accumulation of large quantities of industrial solid waste has exerted considerable pressure on the ecological environment. Under these circumstances, research in the field of construction materials has increasingly focused on the development of environmentally friendly and low-carbon cementitious materials that can partially or fully replace ordinary Portland cement (OPC). The primary objectives are to reduce cement and concrete consumption and to promote the resource utilization of industrial solid waste.

Among various low-carbon cementitious systems, supersulfated cement (SSC) has attracted considerable attention as an alternative to OPC and conventional concrete since the latent hydraulic activity of granulated blast furnace slag (slag) was first identified in 1909 [1]. Owing to its low clinker content and high incorporation of industrial solid waste, SSC offers distinct advantages in reducing carbon emissions [2]. SSC is typically prepared by homogeneously blending slag and gypsum with a small amount of OPC as an activator [3]. Previous studies have demonstrated that SSC enables the high-value utilization of industrial solid waste and that various types of industrial byproduct gypsum can be introduced into SSC as sulfate sources [4,5,6,7,8]. Owing to their wide availability and low cost, industrial byproduct gypsums such as phosphogypsum (PG), titanium gypsum (TG), and flue gas desulfurization gypsum (FGD) have been extensively used in the production of SSC. Among these materials, PG is a bulk solid waste that is generated during the wet-process production of phosphoric acid. It consists predominantly of dihydrate gypsum (DH) with minor amounts of soluble phosphorus, fluorine, and organic impurities [9,10]. Typically, approximately 5 kg of PG is produced for every 1 kg of P_2_O_5_ manufactured [11], and the cumulative stockpile in China has reached approximately 870 million tons [12]. TG is an industrial byproduct of the sulfuric acid process for titanium dioxide production [13]. It is also composed mainly of DH, accompanied by residual Fe_2_O_3_ and TiO_2_ particles [14]. Approximately 9 t of TG is generated per ton of TiO_2_ produced [15]. China produces approximately 18 million tons of TG each year, but only approximately 10% of it is effectively utilized. As a result, the cumulative stockpile has exceeded 130 million tons [16]. FGD is derived primarily from coal-fired power plants and metallurgical processes. Its main component is likewise DH [17], together with certain amounts of calcium carbonate and other impurities [18]. At present, the annual production of FGD in China exceeds 120 million tons and continues to increase at a rate of 15–20% per year [19,20].

Given the large scale of industrial byproduct gypsum resources, their efficient utilization in SSC has become a major focus of recent research. For PG, Wei et al. [21] demonstrated that adjusting the mineral composition of SSC can markedly influence its early-age strength and hydration behavior. Specifically, the incorporation of PG was found to retard early hydration and increase the proportion of fine pores, whereas a synergistic interaction between anhydrite (AH) and PG was observed. Optimizing the proportion of these two sulfate sources was shown to be an effective approach for improving the early mechanical performance of SSC. Gu et al. [8] reported that, in PG-based SSC, partial replacement of granulated blast furnace slag with highly reactive bauxite tailings significantly increased early strength. This improvement was attributed mainly to the promoted formation of ettringite (AFt) and the regulatory effect of aluminum on the structure of C–A–S–H gel. With respect to TG, Xie et al. [4] reported that the incorporation of waste granite stone powder (GSP) effectively regulated the rheological properties and hydration process of TG-based SSC. When the GSP replacement ratio reached 20%, an optimal balance between yield stress and plastic viscosity was achieved, which led to a significant increase in mechanical performance. In contrast, excessive GSP addition (≥30%) resulted in strength deterioration because of insufficient formation of hydration products. With respect to FGD, Ruan et al. [5] reported that partial substitution of slag with low-temperature pretreated FGD significantly accelerated early hydration and improved the mechanical strength of SSC. Shao et al. [6] reported that SSC activated by FGD exhibited high early strength owing to the formation of C–A–H gel. However, the subsequent formation of hannebachite at later ages may hinder strength development.

In summary, previous studies have focused mainly on the role of a single type of industrial byproduct gypsum in SSC; however, systematic comparisons among gypsum sources have been limited. In practice, gypsum from different origins differs markedly in terms of mineral composition, particle characteristics, and dissolution kinetics, which leads to fundamentally distinct sulfate release behaviors within the same SSC matrix. For instance, TG typically contains a certain amount of iron oxide impurities and is characterized by relatively coarser particles, which results in a slower dissolution process. In contrast, FGD typically has finer particles and may introduce soluble impurities such as chloride ions, thereby resulting in more rapid early-age dissolution and ion release. However, how these differences manifest in SSC, as well as the potential synergistic or contrasting effects among different gypsum types, has not yet been systematically clarified. Moreover, in AH–PG-based SSC, the mechanisms by which different gypsums influence sulfate supply, slag activation pathways, and the structural evolution of hydration products remain insufficiently understood.

On the basis of this understanding, this study investigates AH–PG-based SSC and systematically compares the hydration behavior and mechanical performance of AH with those of industrial byproduct TG and FGD in SSC. More importantly, it reveals the intrinsic link between sulfate-release characteristics in different gypsum systems and slag-activation pathways. This work provides new mechanistic insight into the role of byproduct gypsum chemistry in SSC and offers guidance for the performance-oriented design of SSC using diverse industrial gypsums.

## 2. Materials and Methods

### 2.1. Materials

**Figure 1:**
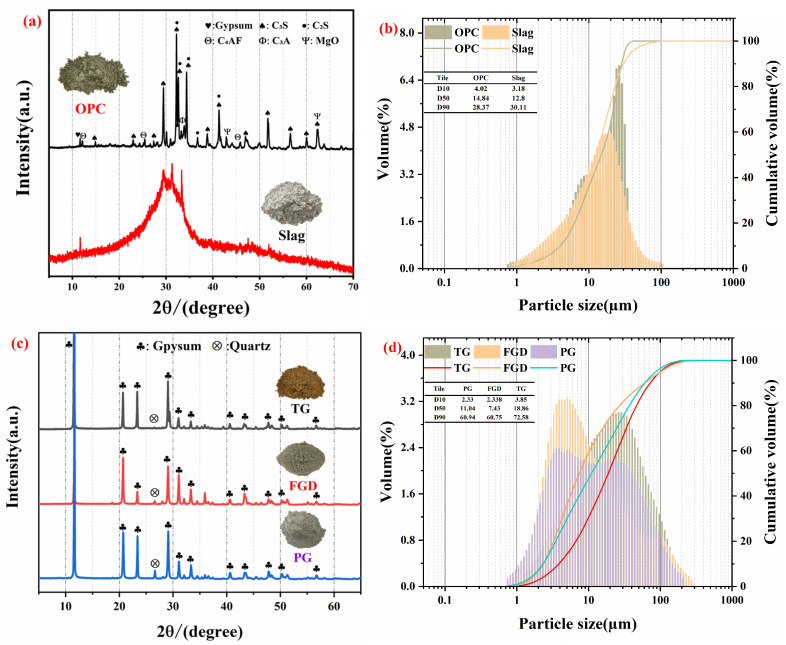
Characterization of raw materials: (**a**) XRD patterns of OPC and slag; (**b**) particle size distribution of OPC and slag; (**c**) XRD patterns of TG, FGD and PG; (**d**) particle size distribution of TG, FGD and PG.

S95-grade slag was obtained from a steel plant in Zhenjiang, Jiangsu Province. PG was supplied by a chemical plant in Jiangsu Province. The OPC complied with Chinese standard GB/T 8076-2008 [22]. AH (CaSO_4_ ≥ 97%) was purchased from Sinopharm Chemical Reagent Co., Ltd. (Shanghai, China). TG was sourced from a TiO_2_ production enterprise in Jiangsu Province. FGD was provided by a power plant in Jiangsu Province. X-ray fluorescence spectroscopy (XRF) was used to characterize the chemical compositions of the selected raw materials, which provide the fundamental basis for understanding the differences in hydration behavior and sulfate supply in the SSC system. The corresponding oxide contents are summarized in Table 1. The results indicated that the slag and OPC were composed primarily of SiO_2_ and CaO, whereas the oxide compositions of PG, TG, and FGD were dominated by CaO and SO_3_. The solubilities, dissolution rates, and retarding effects of different gypsum types are shown in Table 2, where AH refers to soluble AH. X-ray diffraction (XRD) patterns and particle size distribution analyses of the raw materials are presented in Figure 1a–d. Figure 1a shows the XRD patterns of OPC and slag. The slag was mainly composed of an amorphous glassy phase, as indicated by the broad hump in the diffraction pattern, whereas OPC exhibited typical crystalline phases. Figure 1b shows the particle size distributions of OPC and slag, indicating that their particle sizes were primarily in the range of 1–100 μm. Figure 1c presents the XRD patterns of PG, TG, and FGD, in which gypsum was the dominant crystalline phase. Figure 1d shows the particle size distribution characteristics of PG, TG, and FGD. The D_50_ values of OPC, slag, TG, FGD, and PG were 14.84, 12.8, 18.86, 7.43, and 11.04 μm, respectively. Among these materials, TG had the largest median particle size, whereas FGD had the smallest. These differences in particle size are important for the dissolution behavior and sulfate release characteristics of the gypsum materials, which may further affect the hydration kinetics of SSC. In all mixtures, the water-to-binder ratio (W/B) was kept constant at 0.50. Although minor amounts of other oxides were detected by means of XRF, their corresponding crystalline phases were not clearly observed in the XRD patterns, possibly due to their low contents or amorphous nature.

### 2.2. Preparation of Samples

The aim of this study was to systematically investigate the effects of industrial byproduct gypsum, including TG and FGD, on the hydration behavior and mechanical properties of AH–PG-based SSC. Preliminary experiments were conducted to determine the optimal proportions of slag, PG, and OPC [4,23,24]. On the basis of these results, the mixing ratios of AH, TG, and FGD were optimized to enable high-volume utilization of industrial solid waste in the cementitious system. The detailed mix proportions of the SSC samples are listed in Table 3. All raw materials were accurately weighed according to these proportions and thoroughly blended in a mechanical mixer to ensure homogeneity. The resulting mixture was then used as the base binder for subsequent experiments. To investigate the hydration behavior of the system, heat of hydration was measured for a portion of the blended binder using isothermal calorimetry. Mortar samples were prepared with a W/B of 0.50. The binder and standard sand were first mixed uniformly. The mixture was then cast into 40 mm × 40 mm × 160 mm triple steel molds, which was followed by vibration and surface levelling. The samples were cured in a standard curing chamber at 20 ± 1 °C and a relative humidity of at least 90% for 24 h. After demolding, the samples were transferred to water at 20 ± 1 °C and cured for 3, 7, and 28 days prior to mechanical strength testing. For hydration product characterization and fluidity testing, paste samples were prepared using the same binder composition and W/B ratio of 0.50. The paste was stirred until uniform and free of lumps. Fresh paste was either tested immediately for fluidity or sealed in containers and cured at 20 ± 1 °C until the specified ages. Upon completion of curing, the samples were crushed and immersed in absolute ethanol for 24 h to terminate hydration, which was followed by oven drying at 50 °C. The dried samples were subsequently analyzed by means of XRD, thermogravimetric–differential thermal analysis (TG–DTG), Fourier transform–infrared (FT–IR) spectroscopy, mercury intrusion porosimetry (MIP), and scanning electron microscopy (SEM) to characterize the hydration products and microstructure.

### 2.3. Testing Methods

The fluidity of cement paste was determined according to GB/T 8077-2023 [25] using a truncated cone mold, with the flow value taken as the average of two perpendicular diameters after 30 s of spreading.

Hydration heat evolution was monitored by means of isothermal calorimetry (TAM Air C80, TA Instruments, New Castle, DE, USA) at 20.0 ± 0.02 °C for 72 h using sealed paste samples (~4.0 g).

Compressive strength was measured in accordance with EN 196-1:2016 [26] using a microcomputer-controlled electro-hydraulic servo pressure testing machine (HG-YH200F, Jiangsu Zhuoheng Measurement and Control Technology Co., Ltd., Nanjing, China) at curing ages of 3, 7, and 28 days [27].

Phase composition was identified by means of X-ray diffraction (BEDE D1, Bede Scientific Instruments, Durham, UK) using Cu–Kα radiation in the range of 5–70° with a step size of 0.02°.

Thermal behavior was analyzed by means of TG–DTG using a simultaneous thermal analyzer (STA 449F3, NETZSCH, Selb, Germany) from room temperature to 1000 °C at a heating rate of 10 °C min^−1^ under N_2_ atmosphere.

Chemical bonding characteristics were examined by means of Fourier transform–infrared spectroscopy (Nicolet Nexus 670, Thermo Fisher Scientific, Waltham, MA, USA) in the range of 4000–400 cm^−1^.

Pore structure was characterized by means of mercury intrusion porosimetry (AMP-60K-A-1, Porous Materials Inc. (PMI Porometer), Ithaca, NY, USA; China regional distributor: Bailuo (Shanghai) International Trading Co., Ltd., Shanghai, China) within a pressure range of 0.28–415 MPa.

Microstructural features were observed using field-emission scanning electron microscopy (Nova Nano SEM 450, FEI, Hillsboro, OR, USA) after gold sputtering.

The chemical compositions of the raw materials were determined using X-ray fluorescence spectroscopy (S2 PUMA Series II, Bruker, Karlsruhe, Germany).

The particle size distribution of the raw materials was measured using a laser particle size analyzer (BT-9300S, Bettersize Instruments, Shanghai, China).

## 3. Results and Discussion

### 3.1. Fluidity

**Figure 2:**
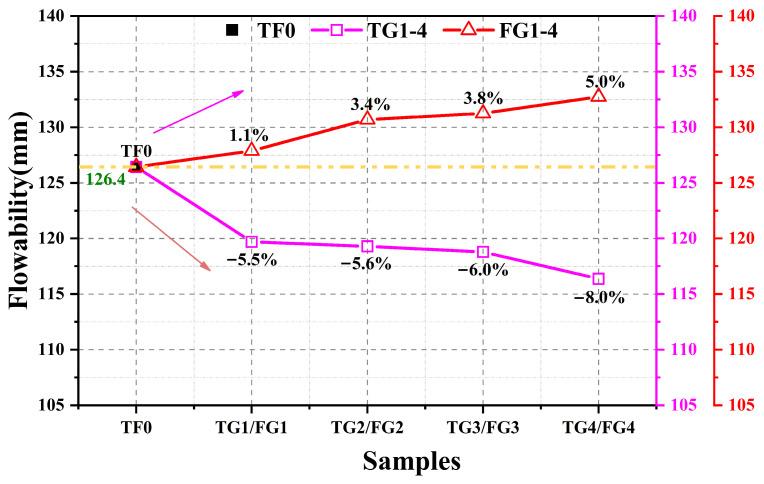
Fluidity of samples.

As shown in Figure 2, TG and FGD had markedly different effects on the fluidity of the SSC. The reference paste had a flow diameter of 126.4 mm. With increasing TG content, the fluidity of the system decreased overall, and the maximum reduction of approximately 8% was observed when TG completely replaced AH. In contrast, increasing the FGD content led to a gradual increase in fluidity, with a maximum improvement of approximately 5% under full replacement of the AH.

These contrasting trends arose mainly from differences in mineral composition and particle characteristics between the two gypsum types. In addition to DH, TG typically contains impurities such as iron hydroxides. These components can reduce paste dispersion by adsorbing dispersants, promoting particle bridging, and consuming free water, thereby lowering fluidity. Moreover, relatively coarse TG particles tend to behave as inert fillers during mixing, which may disrupt the original particle size distribution and form a looser packing structure with higher porosity. As a result, more mixing water is required for pore filling and particle wetting, which decreases the effective fluidity [4]. In contrast, FGD contains SiO_2_ and consists of finer particles, which can effectively fill interparticle voids and reduce friction between solid phases, thereby generating a lubricating effect that enhances the fluidity of the paste [3]. Although the SiO_2_ in FGD does not participate significantly in early-age hydration reactions, its presence mainly influences the fluidity through physical effects, such as increasing particle packing density and modifying the water distribution within the system [4].

### 3.2. Hydration Heat

**Figure 3:**
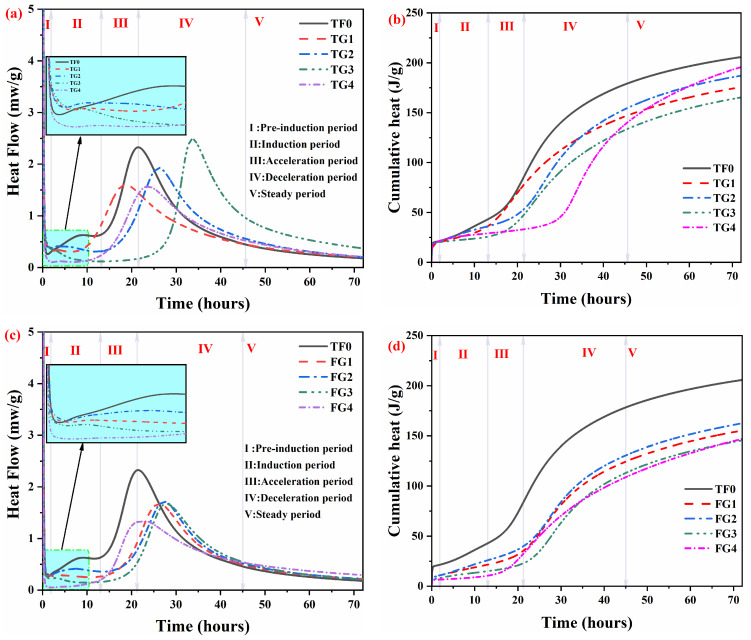
Heat flow and cumulative hydration heat of SSC with TG and FGD. (**a**) Heat flow of TG group; (**b**) cumulative heat of TG group; (**c**) heat flow of FGD group; (**d**) cumulative heat of FGD group.

The heat flow rate and cumulative heat release of the samples within the first 72 h of hydration are presented in Figure 3a–d. According to the hydration process of cementitious systems [4,28], the hydration of SSC can be divided into the pre-induction stage (I), induction stage (II), acceleration stage (III), deceleration stage (IV), and steady stage (V). The hydration heat curves of all of the samples exhibit two dominant exothermic peaks. The first peak appeared in the pre-induction stage and disappeared rapidly during the early hydration period. This peak was associated with the reaction between cement clinker and gypsum, as well as the dissolution of raw materials. At the same time, early hydration products, including AFt, Ca(OH)_2_ (CH), and C–S–H gel, were formed [3,29]. The second peak appeared after the induction stage. This peak corresponded to the activation of slag. Once the concentrations of OH^−^ and Ca^2+^ in the pore solution increased to a level that was sufficient to disrupt the glassy structure of the slag, intensive hydration of the slag occurred [30,31]. In addition, an exothermic peak was observed for samples within the induction period (5–10 h). This behavior was attributed primarily to the rapid dissolution of AH. Owing to the dissolution of AH, the rapid accumulation of SO_4_^2−^ within a short period created a high SO_4_^2−^ environment, which delayed cement hydration and resulted in the formation of a small peak during the induction period [4]. With increasing replacement of AH by TG or FGD, the occurrence time of the small exothermic peak during the induction period in the hydration heat evolution curves was progressively delayed. This indicates that a reduction in AH content weakens the sulfate environment, thereby influencing the hydration process.

In TF0, AH rapidly dissolved during the initial stage and released a large amount of SO_4_^2−^, thereby generating a high-sulfate environment in the pore solution. This environment inhibited cement hydration and the formation of CH, which resulted in a pronounced extension of the induction period. At low TG replacement levels, AH could supply SO_4_^2−^ at early ages. Moreover, the DH contained in TG gradually dissolved and continuously supplied SO_4_^2−^ at the middle and later stages. Consequently, the sulfate release pattern shifted from a single instantaneous release to a combined early–late continuous supply mode. As CH continuously formed and the solution alkalinity was gradually established, a more coordinated dynamic balance between sulfate dissolution and CH formation was achieved, leading to an earlier onset of the induction period. Therefore, compared with TF0, mixtures with low TG replacement exhibited a shortened induction period, which is consistent with the calorimetry results shown in Figure 3a,b.

In the TG system, as the TG replacement increased (TG1–TG3), SO_4_^2−^ was mainly supplied through the dissolution of TG. Under these conditions, the continuously released SO_4_^2−^ from gypsum had a progressively weakened retarding effect on cement hydration, which promoted the formation and accumulation of CH and led to a pronounced establishment of solution alkalinity. However, in the TG, TG2 and TG3 mixtures, these conditions were unfavorable for maintaining an optimal sulfate–CH dynamic balance, which made sufficient activation of the slag at early ages difficult and consequently resulted in a significant prolongation of the induction period. This explains the pronounced prolongation of the induction period observed for the TG2 and TG3 mixtures in Figure 3a. In the TG4 mixture, sulfate supply in the system was governed entirely by TG, and gypsum exhibited stable and continuous dissolution. Compared with systems containing AH, a certain inhibitory effect on early hydration and CH formation was observed. However, in TG4, the absence of rapid, instantaneous sulfate release from AH alleviated this inhibition, thereby promoting the formation and accumulation of CH. As a result, a more favorable sulfate–CH dynamic balance was re-established, leading to a shortened induction period for TG4. Consequently, the induction period of TG4 was significantly shortened compared with those of TG, TG2 and TG3, as clearly reflected in the hydration heat evolution curves.

In the FGD system, the relatively low dissolution rate of DH in FGD weakened the sulfate supply capacity at early ages. The sustained dissolution of FGD also made the system more conducive to maintaining elevated sulfate concentrations compared with the TG system, thereby exerting a stable and long-lasting retarding effect on cement hydration. As the FGD replacement increased from FGD1 to FGD3, the retarding effect gradually intensified, leading to a continuous extension of the induction period. In the FG4 mixture, sulfate was supplied in a stable and continuous manner, which mitigated the strong inhibition of hydration reactions. Consequently, the induction period of FG4 was markedly advanced compared with those of the FGD1–FGD3 mixtures (with partial AH replacement), and was even slightly earlier than that of the TF0 system. Accordingly, the progressive extension of the induction period from FGD1 to FGD3, as well as the advanced induction period of FG4, can be directly attributed to the different sulfate release characteristics of FGD at varying replacement levels.

The enlarged views in Figure 3a,c show that the incorporation of TG and FGD altered the decay rate after the first exothermic peak. At the same replacement level, the post-peak decay rate of the TG system was consistently lower than that of the FGD system. For samples in which AH was partially replaced, the decay rate followed the order: FGD system > control > TG system. When AH was completely replaced, the decay rates of both systems were lower than that of TF0. These differences are related to the dissolution behavior and particle size of the different gypsums [31,32]. As shown in Table 4, TF0 exhibited the highest cumulative heat release after 72 h of hydration, reaching 205.6 J/g. With increasing replacement ratios of AH by TG or FGD, the cumulative heat release of both systems first increased and then decreased. When AH was completely replaced, the cumulative heat release of the TG4 system decreased by 19.7%, while a more pronounced reduction of 28.6% was observed for the FG4 system. These results indicate that the incorporation of TG or FGD reduces the cumulative hydration heat release of SSC, and this effect becomes progressively more significant with increasing replacement levels.

Overall, the sulfate release behavior significantly altered the hydration kinetics of SSC, thereby influencing the early formation of hydration products. These changes in early hydration are expected to directly affect the development of early-age strength, as discussed in Section 3.3.

### 3.3. Compressive Strength

**Figure 4:**
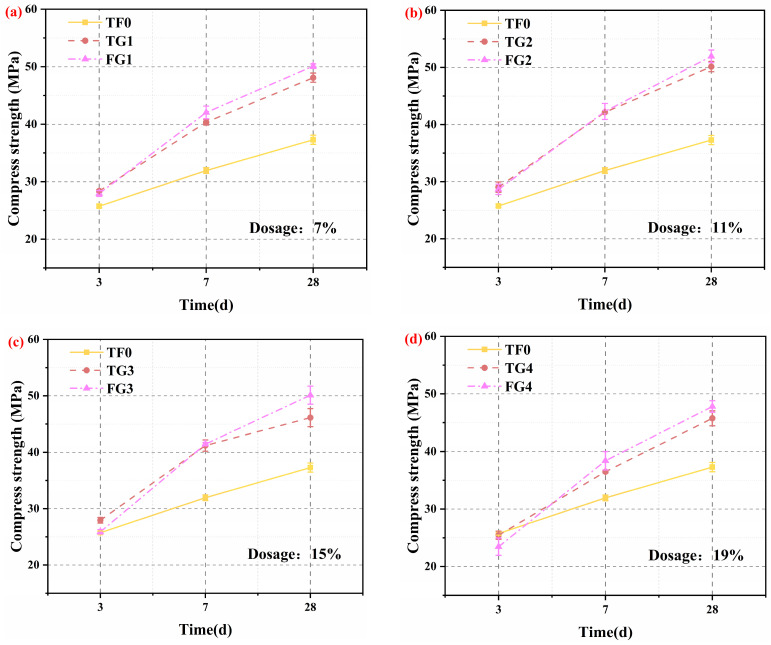
Compressive strength of SSC at curing ages with replacement levels of (**a**) 7%, (**b**) 11%, (**c**) 15%, and (**d**) 19%.

Based on the hydration heat evolution discussed in Section 3.2, the influence of TG and FGD on the mechanical properties of SSC was further evaluated. The effects of various types of gypsum on the compressive strength of SSC at various curing ages under the same dose conditions are shown in Figure 4a–d. It is evident that replacing AH with either TG or FGD significantly altered the strength development pattern.

At 3 days, the compressive strength of the samples that contained TG was greater than that of the samples that contained FGD. However, at 7 and 28 days, the FGD samples outperformed their TG counterparts. This difference can be mainly attributed to the distinct solubility and dissolution behavior of the two gypsum types. TG, with a larger particle size and slower dissolution rate, released SO_4_^2−^ more steadily, thereby leading to a gradual increase in SO_4_^2−^ concentration in the system. This controlled release promoted the formation of a moderate amount of well-crystallized and dense AFt, which provided a stable skeletal framework that increased early-age strength [33,34]. In contrast, FGD has a finer particle size and dissolves rapidly. It released a large amount of SO_4_^2−^ at the early stage. This rapid sulfate supply led to intensive AFt nucleation within a short period. The AFt crystals that formed were finer and loosely packed, thus yielding a weaker C–S–H gel/AFt network and consequently lower early strength. Moreover, an excessively high chloride ion concentration is also unfavorable for the formation of early hydration products and hinders strength development [35]. Nevertheless, the rapid supply of SO_4_^2−^ in the FGD system facilitated more AFt and C–S–H gel formation at later ages, thereby leading to higher strength at 7 and 28 days. When some of the AH remained in the system, compared with the control group, the samples that contained either TG or FGD exhibited higher compressive strength at all ages. This improvement arose from the coexistence of AH and DH, which synergistically regulated SO_4_^2−^ release. This regulation led to more balanced AFt formation and a more stable early-age microstructure, thereby increasing the strength [36]. However, when AH was completely replaced by either gypsum type, the SO_4_^2−^ release pattern was altered. This change resulted in insufficient AFt formation at early ages and a lower 3-day strength than that of the control. With ongoing hydration, AFt and C–S–H gels continued to grow and fill the pore space. The microstructure became denser, which significantly improved strength at later ages.

**Figure 5:**
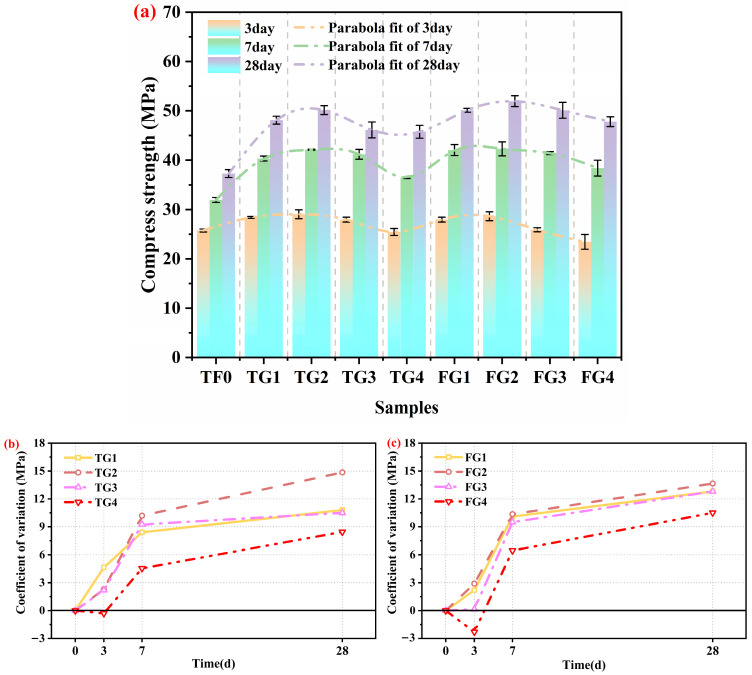
Compressive strength and strength difference of SSC at various curing ages: (**a**) Strength of all gypsum types, (**b**) strength relative to that of FG, and (**c**) strength relative to that of TG.

The compressive strength of the SSC that contained different types of gypsum at various doses, along with the corresponding strength difference relative to the control group, is presented in Figure 5a–c and Table 5. As shown in Figure 5a, within the same gypsum type system, compressive strength exhibits first increased but then decreased with increasing gypsum dose [37]. The strength peaked at a dose of 11%, whereas complete replacement of AH (dose of 19%) resulted in the lowest strength. Specifically, at the 11% dose, the strength of the TG system increased by 12.8% (3 days), 31.9% (7 days), and 34.5% (28 days) compared with that of the control group, whereas the strength of the FGD system increased by 11.3%, 32.4%, and 39.3%, respectively. This trend indicates that an optimal gypsum dose can achieve a favorable balance between the SO_4_^2−^ supply rate and the spatial arrangement of hydration products. Under these balanced conditions, the formation rate and crystal growth of AFt were well coordinated. Moreover, a stable framework was provided for C–S–H gel deposition. As a result, a denser microstructure was formed, leading to improved mechanical performance [38]. In contrast, when the gypsum dose increased to fully replace AH, this balance was disrupted. The synergistic interaction between AFt and the C–S–H gel was weakened. As a result, the microstructure deteriorated. Consequently, the compressive strength decreased.

Considering their performance in terms of hydration heat and compressive strength, TG2 and FG2 were selected as representative samples for subsequent microstructural analyses. The aim of this selection was to provide deeper insights into the effects of TG and FGD on the hydration mechanism and microstructure of AH–PG-based SSC.

### 3.4. XRD Analysis

**Figure 6:**
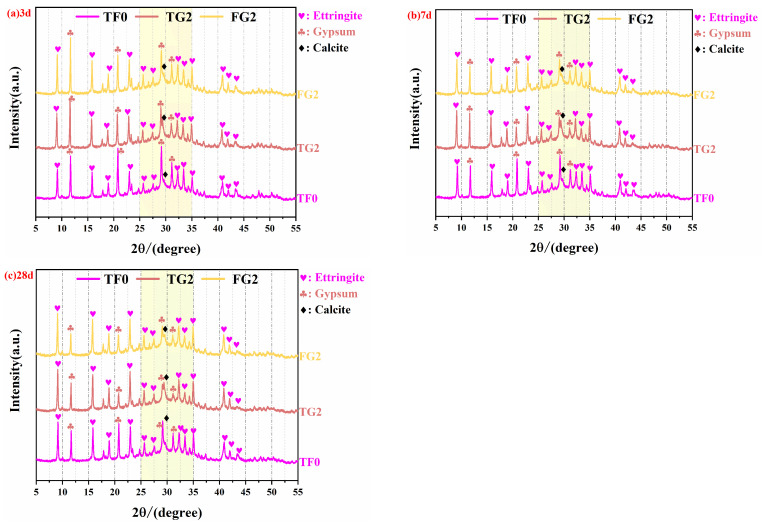
XRD patterns of SSC at various curing ages: (**a**) 3 days, (**b**) 7 days, and (**c**) 28 days.

The XRD patterns of SSC with TG (TG2) and FGD (FG2) at various curing ages are shown in Figure 6a–c. The main hydration products identified include AFt (with characteristic peaks at 9.09°, 15.87°, 18.94°, and 22.94° 2θ) and C–S–H gel. In addition, gypsum (with characteristic peaks at 11.65°, 20.74°, 29.15°, and 30.11° 2θ) and calcite (with a characteristic peak at 29.37° 2θ) were also detected in the patterns. The presence of gypsum can be attributed to unreacted raw materials as well as secondary gypsum formed during the hydration process [39]. Calcite originated primarily from carbonation of the samples through reaction with atmospheric CO_2_, along with a small amount of calcite that was initially present in the raw materials [2]. C–S–H gel, which is an amorphous phase, exhibits a broad hump in the range of 25–35° 2θ. This noncrystalline hump is typically composed of overlapping signals from multiple phases, including C–S–H gel, partial AFt, residual gypsum, calcite, and the amorphous glassy phase of unreacted slag [40,41,42,43].

At a curing age of 3 days (Figure 6a), the AFt characteristic peak intensity of the FG2 sample was the highest, followed by that of TG2, with TF0 showing the lowest value. This indicates that in the FG2 system, gypsum dissolved rapidly in the early stage, which provided an appropriate amount of SO_4_^2−^ to react with the aluminate phase, thereby generating a large amount of AFt. In the TG2 system, the gypsum peak intensity was lower than that in TF0 and FG2, which suggests that the soluble sulfate in the system was consumed more quickly in the early stage, thereby promoting AFt formation [44]. In contrast, the AH in TF0 dissolved rapidly, and excessive SO_4_^2−^ was unfavorable for early AFt formation. Consequently, more residual gypsum remained, which led to a stronger gypsum peak.

At a curing age of 7 days (Figure 6b), the AFt characteristic peak intensities of TG2 and FG2 were comparable. Both were higher than those of the control group. These findings indicate that both types of substituted gypsum can significantly promote AFt formation at this stage. Notably, the intensity of the gypsum peak of FG2 was greater than that of TG2, which suggests that some of the FGD remained unreacted at this age, thus providing a sustained source of sulfate for subsequent hydration. In contrast, the weaker gypsum peak in TG2 implies that most of the soluble sulfate in TG was consumed during the early stage, thereby leading to insufficient sulfate supply in the later stage. The gypsum peak intensity was the greatest in the control group. In contrast, its AFt peak was significantly lower than those of the experimental groups. This observation confirms the effect of excessive SO_4_^2−^ on the formation of hydration products. At a curing age of 28 days (Figure 6c), the AFt peak intensities of TG2 and FG2 remained similar. Both were significantly greater than that of the control group. This finding indicates that both types of substituted gypsum can maintain a high AFt content during long-term curing. The gypsum peak intensity of FG2 was still markedly greater than that of TG2, which reflected a continuous supply of sulfate that facilitated the later-stage hydration of the slag. In contrast, the weaker gypsum peak in TG2 suggests that a limited amount of residual gypsum was available at the later stage. The gypsum peak intensity of the control group was the highest, but the AFt peak was the lowest, which indicates that although a large amount of residual gypsum was present, insufficient early-stage activation led to limited formation of hydration products in the later stage.

In summary, the FG2 system can produce a large amount of AFt in the early stage and maintain a high residual gypsum content in the later stage, thereby continuously stimulating slag hydration. In the TG2 system, sulfate release is relatively uniform in the early stage, and the AFt crystals formed are denser, but the sulfate supply becomes insufficient in the later stage. AH dissolves rapidly, which resulted in limited effects on AFt formation and slag activation. This pattern is consistent with the mechanical performance test results.

The mineral content of the SSC paste was quantitatively analyzed using TOPAS (Version 6.0, Bruker AXS GmbH, Karlsruhe, Germany) software, and the amorphous phase content was calculated via the internal standard method. The quantitative results for individual minerals in the SSC paste were determined using the following equation:

W_rescaled_ = W_Rietveld_ (1 − H_2_O_bound_)

where W_Rietveld_ is the mass fraction of a single mineral phase obtained from the Rietveld refinement in TOPAS software.

**Figure 7:**
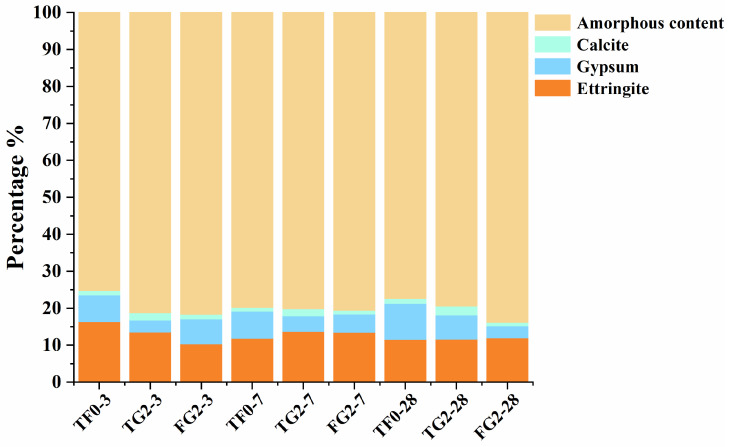
Rietveld refinement results of samples containing TG and FGD at different curing ages.

To better investigate the effects of titanium gypsum and flue gas desulfurization gypsum on the performance of SSC, quantitative phase analysis was conducted on the samples. Figure 7 presents the Rietveld refinement results for the samples at different curing ages. The results indicate that the variation trends of ettringite and gypsum contents at different ages are consistent with the conclusions obtained from the previous compositional and microstructural analyses.

### 3.5. TG–DTG Analysis

**Figure 8:**
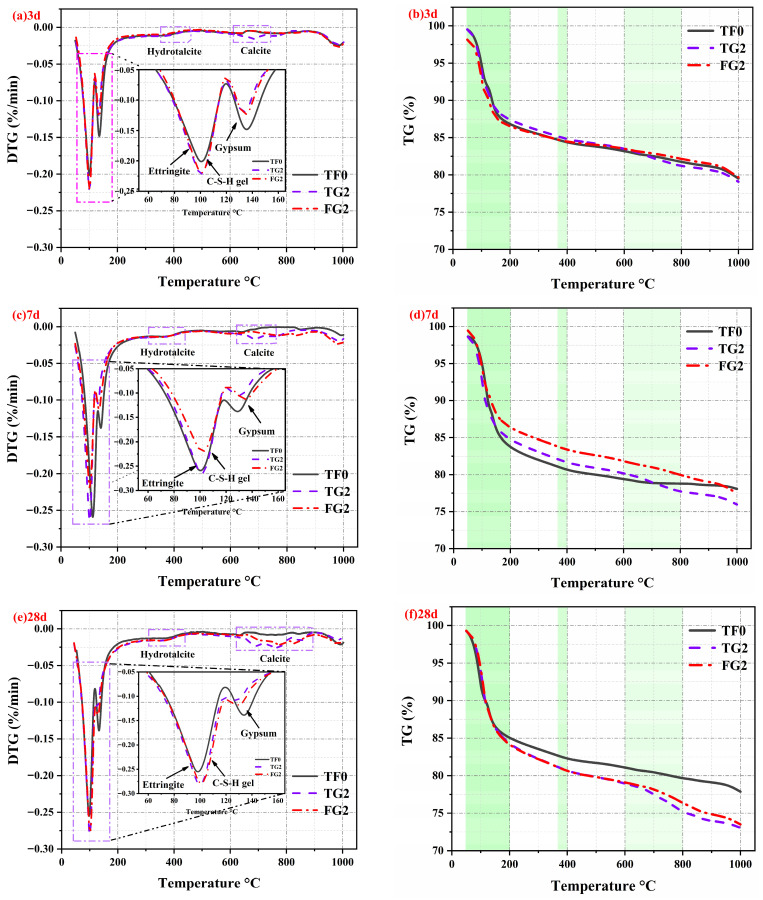
TG–DTG curves of SSC at various curing ages: (**a**,**b**) 3 days, (**c**,**d**) 7 days, and (**e**,**f**) 28 days.

Hydration products were generated during the reaction of SSC with the different types of gypsum. During the heating stage of TG–DTG testing, these products underwent dehydration and decomposition. These processes were accompanied by mass loss and thermal effects. The TG–DTG curves are shown in Figure 8a–f, which correspond to curing ages of 3 days, 7 days, and 28 days, respectively. The DTG results indicate that all samples underwent three main stages of mass loss during heating. The most pronounced peak occurred at 50–200 °C. This peak corresponded to the dehydration of AFt, C–S–H gel, and residual gypsum [45,46]. The peaks observed in the ranges of 360–400 °C and 600–800 °C were associated with the decomposition of hydrotalcite and calcite, respectively. C–S–H gel underwent continuous dehydration in the range of 50–600 °C. Its mass loss overlapped significantly with the dehydration peak of AFt. The mass loss was concentrated mainly at approximately 100 °C. Owing to the complex forms of structural water in C–S–H gel, this process was difficult to identify accurately from the DTG curve [47]. Compared with the TF0 system, part of the AH is replaced by TG or FGD. The peak corresponding to gypsum dehydration in the range of 120–140 °C was significantly reduced. The incorporation of TG or FGD promoted the consumption of gypsum. This accelerated the hydration reaction. It also increased the formation of hydration products. This conclusion is consistent with the results of the XRD analysis.

**Figure 9:**
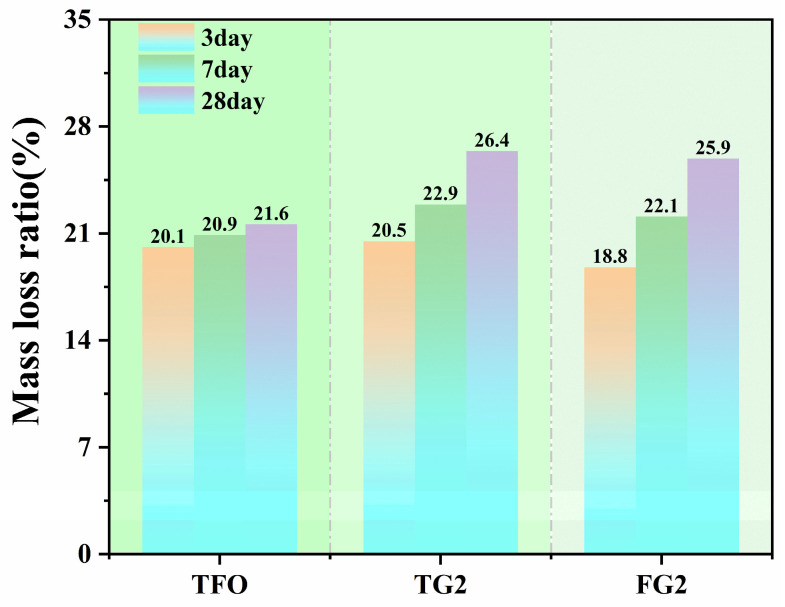
TG–DTG mass loss behavior of SSC from 50 to 1000 °C.

The TG test results at each stage indicate that the mass loss rate gradually increased during the testing process. The mass loss rates of different gypsum-based samples from 50–1000 °C are shown in Figure 9. At 3 days, the mass loss rates of the three samples were 20.1%, 20.5%, and 18.8%. Compared with TF0, the mass loss rate of the FG2 sample was lower. With prolonged curing time, the mass loss rate continued to increase. After 28 days of curing, the mass loss rates of the three samples were 21.6%, 26.4%, and 25.9%, respectively. These results indicate the continuous formation of hydration products. This trend is consistent with the variation in strength.

### 3.6. FT-IR Spectroscopy Analysis

**Figure 10:**
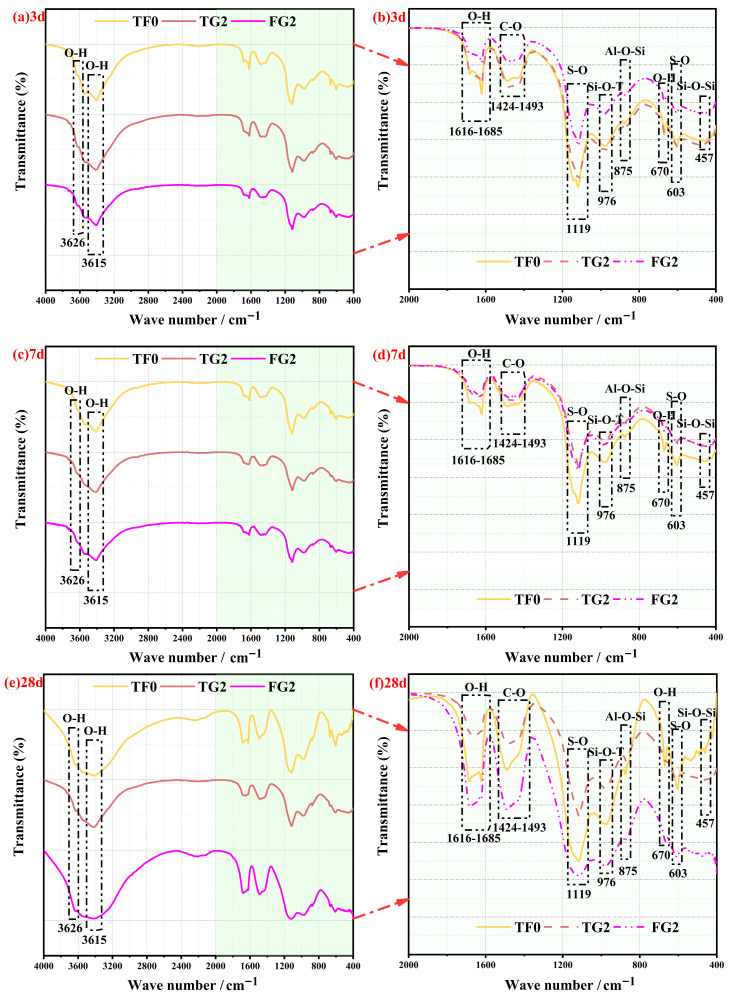
FTIR spectra of SSC at various curing ages. (**a**) 3-day full spectrum; (**b**) 3-day magnified view; (**c**) 7-day full spectrum; (**d**) 7-day magnified view; (**e**) 28-day full spectrum; (**f**) 28-day magnified view.

The FTIR spectra of SSC samples with TG or FGD incorporated at different curing ages for the analysis of hydration products are shown in Figure 10a–f. The major absorption bands remained consistent at all of the curing ages. This finding indicates that replacing AH with different gypsum sources did not introduce new functional groups. According to previous studies, the characteristic bands of gypsum typically appear at 3615–3626 cm^−1^ (asymmetric (ν_3_) O–H stretching and symmetric H–O–H stretching), 1616–1685 cm^−1^ (O–H bending), 603 cm^−1^ (S–O bending), and 1119 cm^−1^ (ν_3_ S–O stretching) [48,49]. These peaks were observed at all of the curing ages in this study, thus confirming the presence of unreacted gypsum. The characteristic absorption bands of AFt occurred mainly in the range of 1616–1685 cm^−1^ and at 1119 cm^−1^. These bands overlapped with those of gypsum, thus indicating that gypsum dissolution played an important role in AFt formation. These observations are consistent with those of previous reports [14,50]. The characteristic bands of C–S–H gel appeared at 976 cm^−1^ (ν_3_ Si–O–T stretching), 875 cm^−1^ (Al–O–Si stretching), and 458 cm^−1^ (Si–O–Si bending). These absorption features have been widely recognized in previous studies as indicators of C–S–H gel formation [14,51]. In addition, an absorption band was observed in the range of 1424–1493 cm^−1^, which corresponded to the ν_3_ vibration of the C–O group. This band originated from the formation of calcium carbonate caused by carbonation of the samples upon exposure to air [52]. After 28 days of curing, the initial doublet that was observed in this region evolved into a single peak. This change was attributed to the transformation of amorphous carbonate into a crystalline phase, such as calcite [4].

### 3.7. MIP Analysis

**Figure 11:**
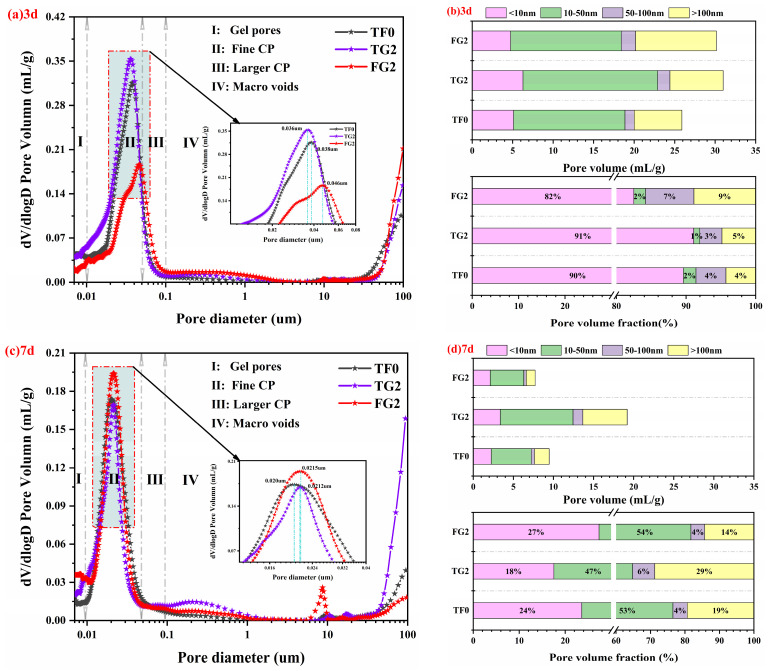

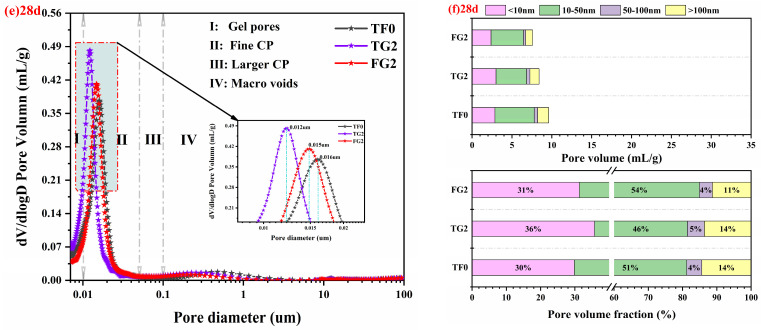
Pore size distribution, pore volume, and pore volume fraction of the SSC. (**a**) 3-day pore size distribution; (**b**) 3-day pore volume and fraction; (**c**) 7-day pore size distribution; (**d**) 7-day pore volume and fraction; (**e**) 28-day pore size distribution; (**f**) 28-day pore volume and fraction.

The effects of incorporating TG or FGD on the pore size distribution, pore volume, and pore volume fraction of SSC samples are shown in Figure 11a–f. The pores in hardened cement paste can be classified into gel pores (<10 nm), fine capillary pores (CP) (10–50 nm), large CP (50–100 nm), and macropores (>100 nm) [53,54]. The properties of the paste were influenced mainly by CP in the range of 10–100 nm [55]. With increasing curing time, the pore distribution gradually shifted from large CP to fine CP. This finding indicates that hydration products continuously filled the pores. As a result, the structure tended to become denser. At all of the considered ages, the critical pore sizes of TG2 were smaller than those of FG2. This may be related to differences in gypsum dissolution behavior and reactivity [21,56,57].

As shown in Figure 11a,c,e, the critical pore sizes of TF0 (containing 19% AH) at 3 days, 7 days, and 28 days were 0.038 μm, 0.020 μm, and 0.016 μm, respectively. For TG2 (in which 11% of the AH was replaced with TG), the values were 0.036 μm, 0.0212 μm, and 0.012 μm, respectively. For FG2 (in which 11% of the AH was replaced with FGD), the values were 0.046 μm, 0.0215 μm, and 0.015 μm, respectively. Compared with TF0, TG2 had smaller critical pore sizes at all ages. At 28 days, the value decreased to 0.012 μm. Compared with TF0, TG2 had smaller critical pore sizes throughout the curing period. This suggests that TG2 can effectively reduce pore connectivity. The continuous formation of C–S–H gel made the microstructure denser and more uniform. At 3 days, FG2 had the largest critical pore size, which was attributed to its fast dissolution rate, leading to loosely packed early AFt and insufficient pore filling. With increasing curing age, the critical pore size decreased to a value comparable to that of TF0. However, the improvement in pore connectivity remained weaker than that of TG2.

As shown in Figure 11b,d,f, the pores in all SSC samples were composed mainly of gel pores and fine CP. At 3 days, the proportion of macropores was greater in FG2 than in TG2, and the pore volume fraction of pores smaller than 100 nm in FG2 was lower than that of TG2 but higher than that of TF0. This phenomenon was attributed to the rapid formation of AFt in the early stage by FGD, which led to a loose structure and uneven pore filling. With increasing curing age, a large amount of C–S–H gel was generated in FG2. The gel occupied the pore space, which led to a significant increase in pores with sizes less than 100 nm. Eventually, this value surpassed those of TG2 and TF0, which indicated a pronounced refinement of the pore structure. In contrast, TG2 released sulfate in a gradual and sustained manner, which led to uniform AFt formation and a stable early pore structure. During the middle and later stages, continued slag hydration densified the matrix. However, the growth rate of the fine pore volume fraction remained lower than that of FG2. These pore structure characteristics are closely related to the mechanical performance of the SSC samples. A reduction in critical pore size and a higher proportion of pores smaller than 100 nm contribute to a denser microstructure and improved load-bearing capacity of the matrix. Therefore, the refined pore structure observed in TG2 and the significant pore refinement at later ages in FG2 are consistent with the compressive strength development of the corresponding samples.

### 3.8. SEM

**Figure 12:**
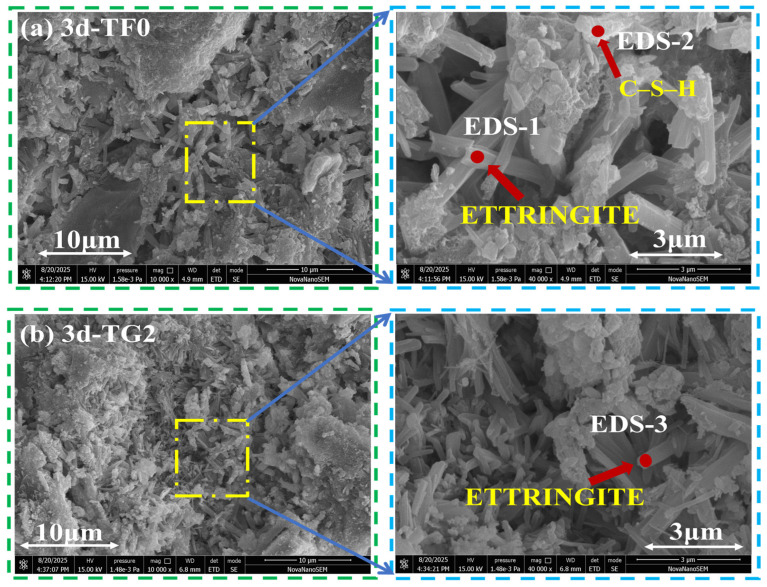

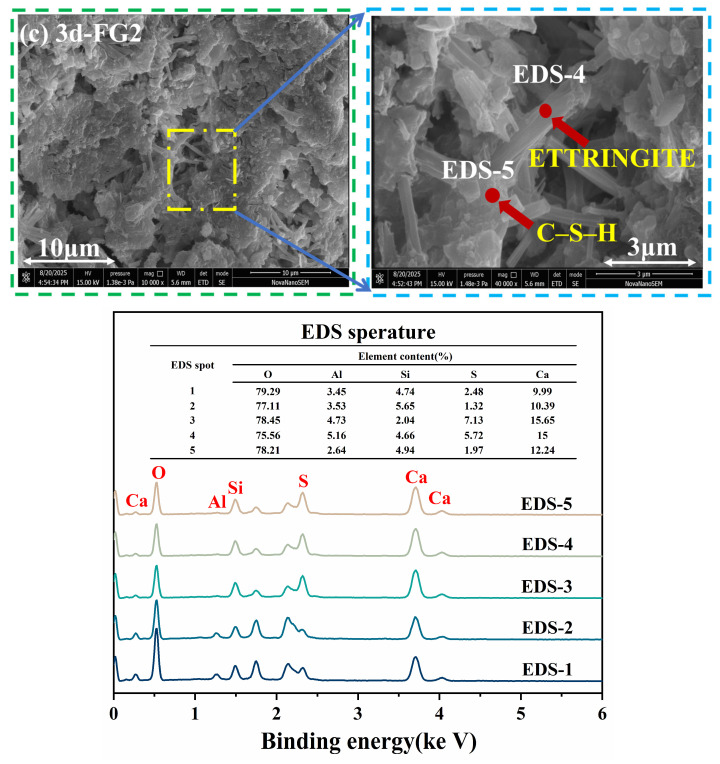
SEM–EDS images of SSC with different gypsum systems at 3 days of curing age. (**a**) TF0; (**b**) TG2; (**c**) FG2.

**Figure 13:**
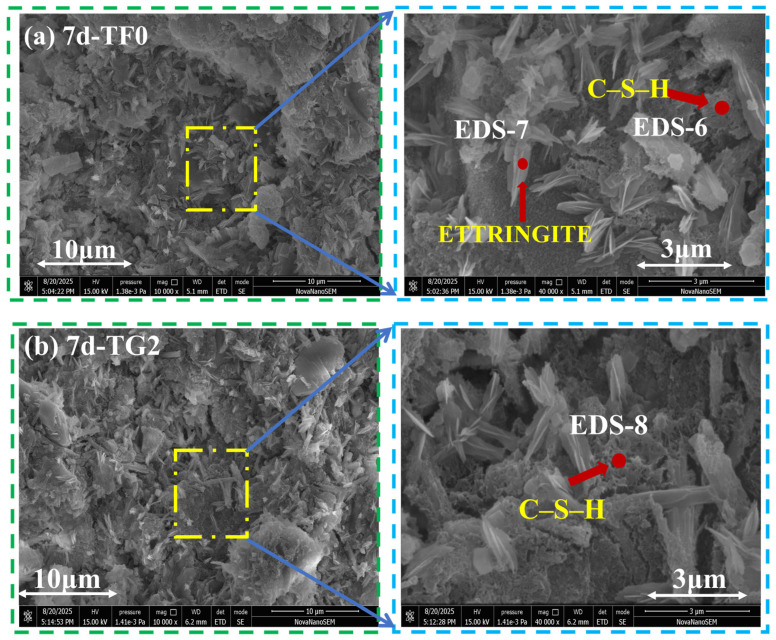

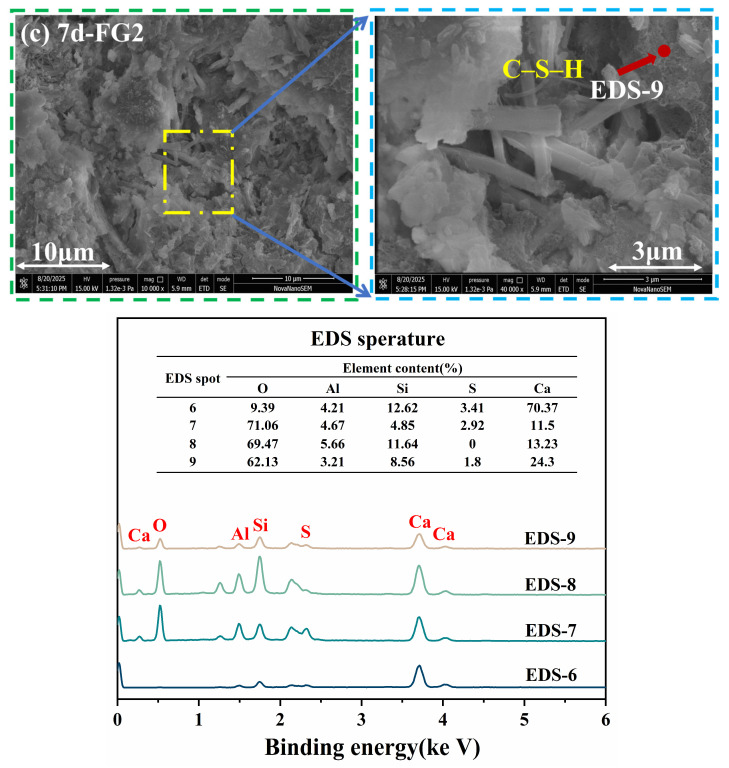
SEM–EDS images of SSC with different gypsum systems at 7 days of curing age. (**a**) TF0; (**b**) TG2; (**c**) FG2.

**Figure 14:**
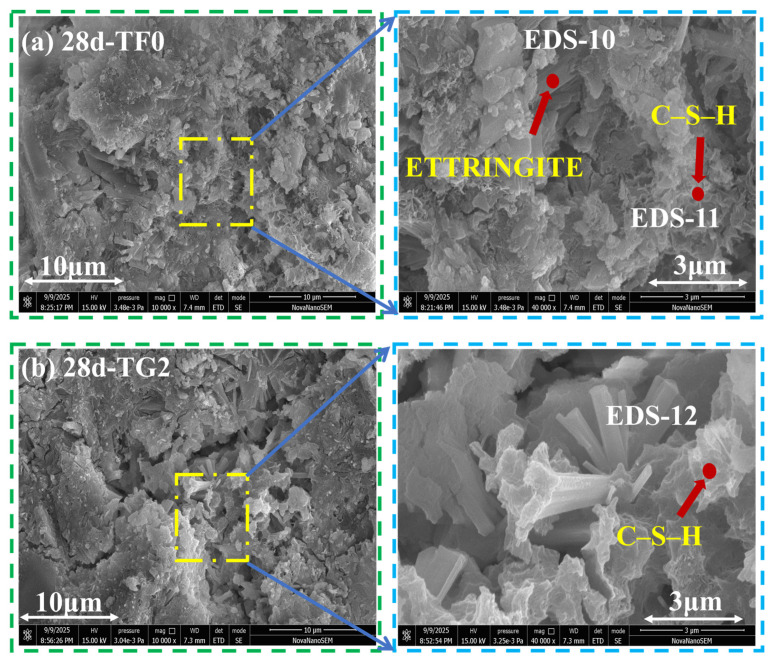

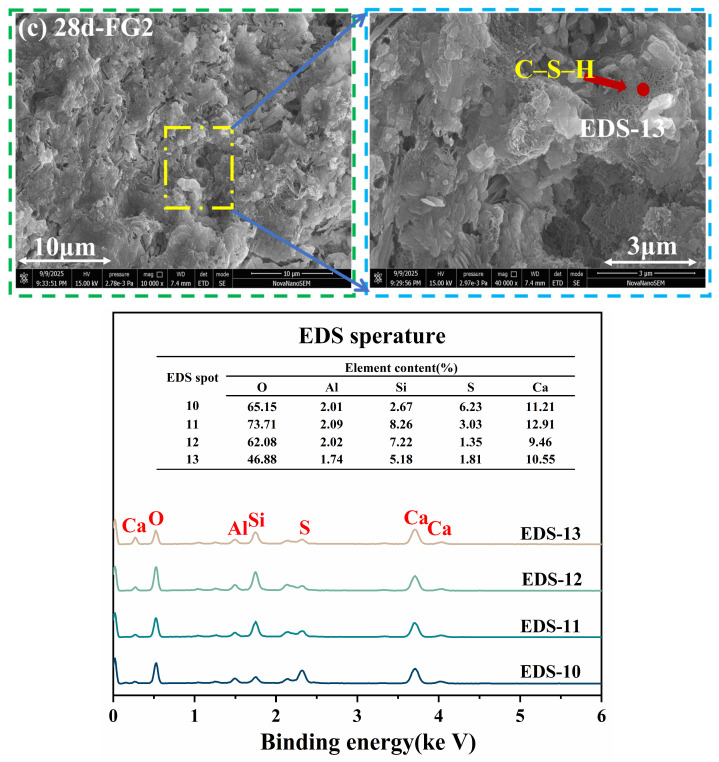
SEM–EDS images of SSC with different gypsum systems at 28 days of curing age. (**a**) TF0; (**b**) TG2; (**c**) FG2.

Figure 12a–c, Figure 13a–c and Figure 14a–c show SEM–EDS images of SSC samples that were prepared with different gypsum systems at curing ages of 3 days, 7 days, and 28 days. EDS analysis indicated that AFt and C–S–H gel constitute the primary hydration products in all the systems. However, their morphology and spatial distribution varied significantly with the type of gypsum and the curing age. At 3 days (Figure 12a–c), the TF0 system showed a large amount of rod-like and needle-like AFt. Aggregated C–S–H gel was also observed within the pores, which resulted in a relatively loose structure. The TG2 system was composed mainly of slender needle-like AFt and small-sized C–S–H gel. The hydration products were more uniformly distributed, and the pore size was relatively small. In contrast, the FG2 system formed large blocky AFt and gel products. Their loose stacking led to a pronounced increase in pore size.

At 7 days (Figure 13a–c), both the TF0 and TG2 systems showed large amounts of growing C–S–H II gel and typical honeycomb-like C–S–H gel structures [58]. This indicated that the pores were gradually filled by hydration products. In contrast, the FG2 system was still dominated by aggregated C–S–H gel and rod-like AFt. The hydration products are distributed less uniformly. At this stage, the pore size retained between hydration products in the TG2 system is generally larger than that in the FG2 system.

At 28 days (Figure 14a–c), the pore structure of all systems became further densified, and obvious pores were located mainly in local microcrack regions. In the TF0 system, a honeycomb-like C–S–H gel was observed together with a small amount of AFt. In the TG2 system, rod-like AFt was enriched inside the cracks and was wrapped by aggregated C–S–H gel. A dense structure formed around the cracks. In the FG2 system, the number and size of the cracks were relatively small. AFt was wrapped by C–S–H gel, and the structure inside the cracks was relatively compact. Overall, differences in the formation of AFt and the filling behavior of C–S–H gel among the gypsum systems directly led to different pore structure evolution paths. The observed morphological features were in good agreement with the MIP results and the development of mechanical performance.

## 4. Hydration Mechanism Analysis

**Figure 15:**
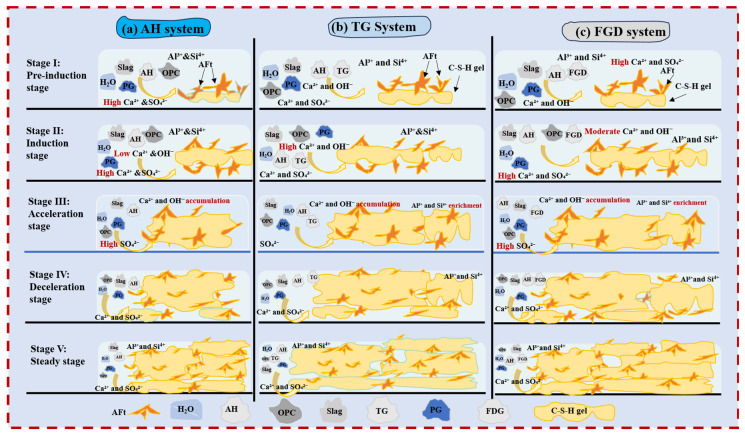
Hydration mechanism of SSC with various gypsum systems. (OPC: ordinary Portland cement; TG: titanium gypsum; FGD: flue gas desulfurization gypsum; PG: phosphogypsum; AH: anhydrite; AFt: ettringite; C–S–H: calcium silicate hydrate).

**Figure 16:**
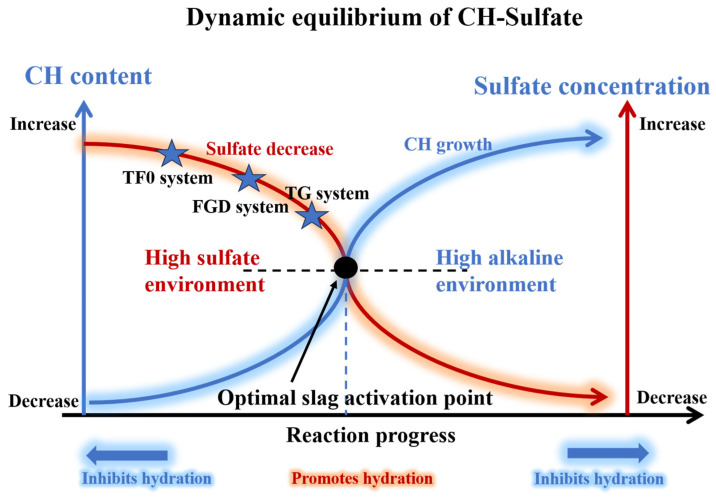
CH–sulfate dynamic equilibrium. (TG: titanium gypsum; FGD: flue gas desulfurization gypsum; CH: calcium hydroxide).

The hydration mechanism of an AH–PG-based SSC under different gypsum systems is shown in Figure 15. The hydration processes in the three systems can generally be divided into raw material hydration or dissolution, sulfate regulation, slag activation, and microstructural densification. However, differences in gypsum type, particularly in terms of solubility, result in pronounced variations in the dominant reactions at each stage. The dynamic equilibrium relationship between CH and sulfate is shown in Figure 16.

(1) AH system

After contact with water, OPC, PG, and AH rapidly undergo hydration and/or dissolution reactions. Among them, AH quickly hydrates to form DH in the presence of water and subsequently dissolves, which results in the release of large amounts of Ca^2+^ and SO_4_^2−^ into the system. Owing to the high dissolution rate of AH, SO_4_^2−^ rapidly accumulates at the early stage, thus establishing a high sulfate level during the induction period and giving rise to a pronounced exothermic peak associated with gypsum dissolution. In this high-sulfate environment, cement hydration is significantly retarded, and the formation and effective accumulation of CH are restricted. Consequently, the development of system alkalinity is delayed, which leads to a prolonged induction period and a corresponding postponement of the slag reaction. As hydration proceeds, CH gradually forms and accumulates, and the system alkalinity eventually reaches the level required to activate the slag. The slag then undergoes intensive hydration, which is accompanied by the formation of large amounts of hydration products. At a later stage, AFt develops a spatial skeletal framework, and C–S–H gel continuously fills the pores, thereby progressively optimizing the pore structure and promoting system densification.

(2) TG system

In the TG system, gypsum exists mainly in the form of DH, which is characterized by a relatively large particle size and a lower dissolution rate (with a particle size larger than that of FGD and a dissolution rate lower than that of AH). Upon water addition, OPC, PG, AH, and TG simultaneously undergo hydration and/or dissolution reactions, and their kinetic behaviors vary markedly with respect to the TG replacement ratio. At low TG replacement levels, a relatively high proportion of AH is still retained in the system, which can rapidly release SO_4_^2−^ at the initial mixing stage, whereas TG provides a sustained sulfate supply at later ages through gradual dissolution. As a result, the sulfate supply mode progressively shifts from a single “instantaneous release” to a combined “early–late continuous release,” and the sulfate release rate gradually becomes better matched with the generation rate of CH during cement hydration. With the continuous formation of CH, the alkalinity of the solution is progressively established, and a near-optimal dynamic balance is achieved between sulfate dissolution and CH formation. Consequently, the system breaks through the induction period at an earlier stage, as evidenced by a pronounced shortening of the induction period and an earlier onset of the acceleration period. With an additional increase in the TG replacement ratio, the AH content decreases significantly, which weakens the ability of the system to rapidly release SO_4_^2−^. Under these conditions, the continuously released SO_4_^2−^ from gypsum has a progressively weakened retarding effect on cement hydration, which promotes the formation and effective accumulation of CH and leads to pronounced solution alkalinity. However, such conditions are unfavorable for maintaining an optimal sulfate–CH dynamic balance, which makes sufficient activation of the slag at early ages difficult and consequently results in a significant prolongation of the induction period. When the AH was completely replaced by TG, sulfate supply in the system was governed entirely by TG, and gypsum exhibited stable, continuous dissolution behavior. Compared with AH-containing systems, the transient high-sulfate environment that was caused by the rapid dissolution of AH strongly inhibited early hydration as well as the formation and effective accumulation of CH. In contrast, in the pure TG system, the absence of instantaneous sulfate release from AH significantly alleviated the inhibition of early hydration, thereby promoting CH formation and accumulation. Consequently, a more favorable sulfate–CH dynamic balance was re-established, which resulted in a markedly shortened induction period for the pure TG system compared with systems with high TG replacement levels, with a duration that approached that of the TF0 system. Once the slag is sufficiently activated, the system enters an intensive hydration stage and produces large amounts of AFt and C–S–H gel. In the later stage, AFt forms a continuous skeletal framework, and C–S–H gel fills the pores, which ultimately leads to the development of a dense and stable microstructure. In the later stage, AFt forms a continuous skeletal framework. The C–S–H gel fills the pores. This process ultimately leads to the formation of a dense and stable microstructure.

(3) FGD system

In the FGD system, gypsum exists predominantly in the form of DH, which features relatively fine particles (smaller than those of TG) and a dissolution rate that is lower than that of AH. After water addition, OPC, PG, AH, and FGD simultaneously undergo hydration and/or dissolution reactions. Because the dissolution rate of FGD is markedly lower than that of AH, the capacity of the system for rapid SO_4_^2−^ supply at early ages is significantly weakened. Moreover, SO_4_^2−^ from FGD continuously enters the solution via sustained dissolution, thereby maintaining a relatively high sulfate level in the system. During the stage in which FGD partially replaces AH, the substantially lower dissolution rate of FGD reduces the rapid early supply of SO_4_^2−^. Moreover, the continuous dissolution of FGD persistently replenishes SO_4_^2−^ in the solution, which enables the system to remain at a relatively high sulfate level over an extended period. The sustained presence of SO_4_^2−^ retards cement hydration, thereby restricting the formation and effective accumulation of CH. As the FGD replacement ratio increases, this inhibitory effect becomes progressively more pronounced and results in a corresponding extension of the induction period. When AH is completely replaced by FGD, the instantaneous high-sulfate release behavior is likewise eliminated, and sulfate supply shifts from a transient release mode to a stable and continuous supply mode. In AH-containing systems, the rapid dissolution of AH produces a transient high-sulfate environment at early ages, which significantly inhibits cement hydration and the formation and effective accumulation of CH. In contrast, in the pure FGD system, the absence of AH-induced instantaneous sulfate release substantially reduces the inhibitory effect on early hydration, which is more conducive to CH formation and accumulation. Consequently, a relatively favorable sulfate–CH dynamic balance is re-established, which causes the induction period of the pure FGD system to occur markedly earlier than that of partially FGD-replaced systems and even earlier than that of the TF0 system. With the continuous accumulation of OH^−^ and Ca^2+^ in the system, the glassy structure of slag gradually disintegrates and the slag becomes effectively activated. In the later stage of the reaction, large amounts of AFt progressively form a spatial skeletal framework, and C–S–H gel continuously forms, fills the pores, and interweaves, which leads to ongoing pore structure refinement and ultimately the formation of a relatively dense and stable microstructure.

## 5. Conclusions

In this study, the effects of TG and FGD on the hydration behavior, fluidity, mechanical properties, and microstructural evolution of an AH–PG-based SSC were systematically examined. By adjusting the dissolution rate and solubility of gypsum, the formation rate of calcium hydroxide can be modified, thereby effectively regulating the reaction process of SSC and enabling the controlled preparation of high-strength SSC. The research findings provide a theoretical basis for the efficient utilization of various industrial byproduct gypsums and offer important guidance for the controllable design of SSC performance. On the basis of a systematic experimental investigation of the hydration process and mechanical performance, the following conclusions are drawn:

1. TG and FGD have opposite effects on SSC fluidity. TG reduces fluidity because of impurity effects and coarse particles that weaken particle packing. FGD increases fluidity because its fine particles fill voids and provide a lubricating effect.

2. The incorporation of TG and FGD significantly alters the early hydration heat characteristics of SSC by mitigating the inhibitory effect caused by the rapid dissolution of AH and the resulting high sulfate concentration, thereby optimizing the early reaction.

3. With increasing AH replacement levels, the cumulative heat release of the system decreases progressively. Under complete replacement conditions, the cumulative heat release was reduced by approximately 19.7% for the TG system and 28.6% for the FGD system.

4. TG increases early-age (3 days) strength, whereas FGD yields higher late-age (7–28 days) strength. An optimal gypsum dose of approximately 11% yielded the highest compressive strength. The coexistence of AH and DH has a synergistic effect on sulfate availability, which leads to a denser and more stable microstructure.

5. Both TG and FGD significantly refine pore structure with increasing curing age. TG consistently yields smaller critical pore sizes and denser microstructures, whereas FGD has a looser early structure but pronounced later refinement, consistent with strength trends.

6. Microstructural analyses confirmed that the type of gypsum plays a decisive role in regulating AFt formation, residual gypsum consumption, and pore structure refinement. Variations in particle size distribution and dissolution kinetics among different gypsum samples determine the sulfate release characteristics and slag activation mechanisms, thereby leading to distinct hydration pathways, pore structure evolution, and microstructural densification processes.

In addition to the microstructural and mechanical improvements, the environmental implications of this study should be emphasized. SSC is recognized as a low-carbon alternative to ordinary Portland cement due to its reduced clinker content and extensive utilization of industrial byproducts. The incorporation of TG and FGD further promotes the large-scale recycling of industrial waste gypsum, thereby contributing to resource efficiency and waste minimization. By enabling controlled regulation of hydration and strength development through gypsum type selection, this study provides practical guidance for the sustainable and industrial-scale production of high-performance SSC. Therefore, the developed system demonstrates significant potential for carbon emission reduction and green construction applications.

## Figures and Tables

**Table 1:** Compositions of raw materials as determined by means of XRF.

Material	Mass Fraction of Each Chemical Component/%										
	Al_2_O_3_	SiO_2_	Na_2_O	MgO	CaO	Fe_2_O_3_	K_2_O	SO_3_	TiO_2_	Cl^−^	LOI
Slag	14.08	32.69	0.55	6.63	37.18	-	5.03	1.56	1.23	0.04	1.01
OPC	3.83	17.62	0.23	2.74	65.3	3.24	0.92	3.65	0.31	0.02	2.14
TG	1.68	2.03	0.24	0.91	27.97	6.23	0.18	19.72	2.69	0.06	38.29
FGD	2.46	6.51	0.11	0.17	24.39	0.3	0.21	21.08	0.29	0.37	44.11
PG	1.34	4.49	0.74	0.12	31.87	-	0.14	29.09	0.07	-	32.14

**Table 2:** Solubilities, dissolution rates, and retarding effects of various gypsum types.

Gypsum Type	Solubility (g/L)	Relative Dissolution Rate	Relative Retarding Effect
Soluble anhydrite	6.0	Fast	Very strong
Dihydrate gypsum	2.4	Slow	Strong
Hemihydrate gypsum	6.0	Fast	Very strong
Natural anhydrite	2.1	Very slow	Weak

**Table 3:** Mix proportions of the samples (%).

Samples	Cementitious Material (%)						Water (g)	Sand (g)
	Slag	OPC	PG	AH	FGD	TG		
TF0	72	5	4	19	0	0	225	1350
TG1	72	5	4	12	0	7	225	1350
TG2	72	5	4	8	0	11	225	1350
TG3	72	5	4	4	0	15	225	1350
TG4	72	5	4	0	0	19	225	1350
FG1	72	5	4	12	7	0	225	1350
FG2	72	5	4	8	11	0	225	1350
FG3	72	5	4	4	15	0	225	1350
FG4	72	5	4	0	19	0	225	1350

**Table 4:** Cumulative heat of hydration of SSC with TG, FGD, and without TG/FGD at different time intervals (J/g).

Hydration Time/h	Blank	TG				FG			
	TF0	TG1	TG2	TG3	TG4	FG1	FG2	FG3	FG4
1	20.6	18.9	20.3	20.1	20.3	10	9.9	7.5	6.6
2	21.5	20.1	21.6	20.5	21.7	11	10.9	8.3	6.8
3	22.8	21.3	23	20.9	22.9	12	12.1	9.1	6.9
6	27.6	24.8	27.4	22.2	25.6	15.1	16.2	11.3	7.6
12	40.7	33.3	34.9	24.9	28.6	20.6	24.7	14.6	9.9
24	105.9	89.9	65.9	61.5	34.9	47.3	49.8	30.6	45.5
48	182.9	150.9	159.3	138	148	129.5	135.8	118.6	114.4
72	205.8	175.6	187.1	165.2	195.8	155.3	162.5	145.4	146.9

**Table 5:** Compressive strength and strength difference of SSC.

Sample	Average Compressive Strength (MPa)			Strength Difference ^a^		
	3 Days	7 Days	28 Days	3 Days	7 Days	28 Days
TF0	25.75	31.95	37.30	0	0	0
TG1	28.40	40.35	48.10	4.65	8.40	10.80
TG2	29.05	42.15	50.15	2.30	10.20	12.85
TG3	27.95	41.20	47.80	2.20	9.25	10.50
TG4	25.45	36.50	45.75	−0.30	4.55	8.45
FG1	27.95	42.05	50.10	2.20	10.10	12.80
FG2	28.65	42.30	51.95	2.90	10.35	14.65
FG3	25.90	41.45	50.10	0.15	9.50	12.80
FG4	23.45	38.40	47.80	−2.30	6.45	10.50

^a^ Strength difference calculated as the difference between the tested mixture and the control.

## Data Availability

The original contributions presented in this study are included in the article. Further inquiries can be directed to the corresponding authors.
